# Vitamin B_12_ Availability and Interaction with Bacterium *Mesorhizobium loti* Affect Growth and Biochemical Composition of Mixotrophic Alga *Euglena Gracilis*

**DOI:** 10.1007/s00248-026-02816-0

**Published:** 2026-06-23

**Authors:** Sadikshya Ghimire, Mohammad Salar Sohrabi, Xuan Zhou, Marjo Malinen, Markku Keinänen, Steffi Goffart, Sarita Keski-Saari, Ursula Strandberg

**Affiliations:** 1 Department of Environmental and Biological Sciences, University of Eastern Finland, Joensuu, 80101 Finland; 2 Department of Forestry, Food Technology, and Environmental Technology, South-Eastern Finland University of Applied Sciences, Kouvola, 45100 Finland; 3 Centre for Photonics Sciences, University of Eastern Finland, Joensuu, 80101 Finland

**Keywords:** Microalgae, Vitamin B_12_, PUFAs, DOC, Proteins, EPA, DHA

## Abstract

Over 90% of the mixotrophic algae surveyed require vitamin B_12_ (B_12_), a micronutrient synthesized exclusively by certain prokaryotes. Bacteria-mediated B_12_ supply presents an important but poorly understood control on physiology and nutritional quality of freshwater algae. Here, we examined the effects of B_12_ availability and the B_12_-synthesizing bacterium, *Mesorhizobium loti*, on the growth and biochemical composition of the freshwater bloom-forming mixotrophic alga, *Euglena gracilis*. We also investigated bacteria-alga interactions under highly labile dissolved organic carbon (DOC) supplementation with and without physical contact. Our results indicate that B_12_ deficiency reduced algal growth and the accumulation of the essential fatty acids, eicosapentaenoic acid (EPA) and docosahexaenoic acid (DHA). Co-culture with *M. loti* resulted in higher EPA and DHA accumulation than supplementation with exogenous B_12_ alone. Despite this, *E. gracilis* accumulated less intracellular B_12_ in co-culture than under direct B_12_ addition, suggesting altered B_12_ acquisition or utilization during bacterial interactions. Increasing B_12_ concentration enhanced total protein content in *E. gracilis* under sufficient nitrogen conditions but had no effect under nitrogen limitation. The addition of labile DOC directly promoted algal growth, indicating its direct utilization. No significant difference was observed in algal growth in co-cultures with and without physical contact, indicating that possible B_12_ sharing from bacteria mainly occurs through diffusible metabolites. These findings demonstrate that B_12_-synthesizing bacteria influence the growth and nutritional quality (in terms of EPA, DHA and protein content) of *E. gracilis*, with potential consequences for freshwater ecosystem productivity and trophic transfer.

**Supplementary Information:**

The online version contains supplementary material available at https://doi.org/10.1007/s00248-026-02816-0.

## Introduction

Cobalamin, also known as vitamin B_12_ (hereafter B_12_), is an important vitamin that acts as a growth factor in about 50% of eukaryotic algae [1–3]. B_12_-independent eukaryotic algae do not require B_12_ for growth, yet they can assimilate B_12_ when available, highlighting the fundamental role of B_12_ for phytoplankton productivity and nutrient cycling [4]. In fact, reduced B_12_ availability has been shown to constrain phytoplankton production in marine ecosystems [5–7]. The synthesis of B_12_ is limited to certain bacteria and archaea [8]. Thus, B_12_ auxotrophic algae, i.e., algae that require B_12_ for growth but lack the capacity for *de novo* synthesis, rely on external sources of B_12_. These auxotrophic algae acquire vitamin B_12_ primarily through two pathways: direct uptake of dissolved B_12_ released by B_12_-synthesizing bacteria or via symbiotic interaction with these bacteria [1, 9–11]. The interactions between B_12_-synthesizing bacteria and B_12_-auxotrophic algae are most likely modulated by environmental conditions [12]. For instance, the mutual exchange of B_12_ and carbohydrates between algae and bacteria can be disrupted by alternative carbon sources [2].

Algae exhibit diverse resource acquisition strategies. Some algae are strictly autotrophic, relying on photosynthesis to produce organic carbon, whereas others are mixotrophs, combining photosynthesis with heterotrophy, such as phagotrophy, i.e., the ingestion of prey such as bacteria, to meet their nutritional requirements [13–16]. By integrating these two strategies, mixotrophic algae often dominate freshwater phytoplankton communities and frequently form blooms [17]. Their dual nutritional mode also provides a competitive advantage in environments with low-light availability and high bacterioplankton biomass, where they can acquire macronutrients and essential trace elements through the ingestion of bacterial prey [15, 18]. More than 90% of surveyed mixotrophic algae require B_12_ for growth [10]. Therefore, B_12_-synthesizing bacteria are expected to play a key role in regulating the dynamics of mixotrophic algal communities. While mutualistic interactions between B_12_-auxotrophic algae and B_12_-synthesizing bacteria have been tested on autotrophic algae, our understanding of such interactions in mixotrophic algae remains limited. In particular, it is unclear to what extent phagotrophy contributes to B_12_ acquisition in mixotrophic algae.

B_12_ is a cofactor for methionine synthase, an enzyme involved in one-carbon metabolism, influencing, among others, lipid biosynthesis. B_12_ availability may affect the biosynthesis of essential polyunsaturated fatty acids (PUFAs), such as eicosapentaenoic acid (EPA) and docosahexaenoic acid (DHA), in algae. EPA and DHA are essential for many aquatic animals, and B_12_-mediated biosynthesis of EPA and DHA at the base of the food chain influence EPA and DHA levels throughout the food web [32]. Additionally, methionine synthase is linked to nitrogen assimilation and protein synthesis in algae [19]. The effects of B_12_ deficiency on synthesis of PUFA and proteins in algae are still poorly known.

This study aims to investigate the effects of B_12_ availability and the abundance of B_12_-synthesizing bacterium (*Mesorhizobium loti*) on the growth and the content of fatty acids (EPA, DHA) and proteins of *Euglena gracilis* under varying nitrogen conditions. Co-cultivation of *E. gracilis* with bacterium *Pseudobacillus badius* supplied vitamin B_12_ for long term growth of *E. gracilis* [20]. Similarly, biomass and lipids production by *E. gracilis* was enhanced in co-culture with bacterium *Emticicia sp. EG3* [21]. *E. gracilis* may form dense blooms in eutrophic conditions but is not known to produce toxins. However, dense blooms on the surface of water deteriorate water quality, shade other photosynthetic producers, and crashing blooms may cause hypoxia. *E. gracilis* is a B_12_ auxotrophic, flagellated, mixotrophic algae, which is rich in chlorophyll a [22, 23]. It synthesize polysaccharide β-1,3-glucan called paramylon [24, 25], polyunsaturated fatty acids (PUFAs) such as EPA and DHA [23], and is rich in proteins [26]. It is auxotrophic for vitamin B_12_, thus requires exogenous supply of this cofactor. Interaction with B_12_ producing bacteria may play a crucial role in growth and productivity of this alga.

In addition, we investigated the effects of a labile dissolved organic carbon (DOC) supplementation and restricted direct physical contact between *E. gracilis* and *M. loti* on algal growth dynamics. *M. loti* is a heterotrophic soil bacterium belonging to the order Rhizobiales and is known to synthesize B_12_ [2, 10]. We hypothesize that (i) increased B_12_ availability would enhance the growth rate, total protein, and fatty acid content of *E. gracilis;* (ii) co-limitation of nitrogen and B_12_ decreases protein content in *E. gracilis;* (iii) *E. gracilis* grows better when co-cultured with *M. loti* than when supplied with external B_12_ as bacteria may release other growth promoting substances along with B_12_, and (iv) physical contact with B_12_-synthesizing bacterium further promotes *E. gracilis* growth.

## Materials and Methods

### Microbes and Growth Conditions

The mixotrophic alga *Euglena gracilis* (NIVA-1/79) was purchased from Norwegian Culture Collection of Algae (NORCCA), based at the Research Institute for Water and the Environment (NIVA). The bacterium *Mesorhizobium loti* (HAMBI:1131) was purchased from the HAMBI culture collection at the University of Helsinki, Finland. Prior to the experiments, the algal cultures were treated with a penicillin-streptomycin mix (Sigma-Aldrich) containing 10,000 units of penicillin and 10 mg mL^− 1^ streptomycin. Purity of algal subcultures were verified by plating on three different agar plates (nutrient, LB and TY) for 48 h at 25 ℃ prior to the experiment. Cultures were regularly monitored using an inverted microscope to screen for any bacterial presence. Algal cultures were maintained in modified CW-15 (MCW-15) medium according to the NORCCA protocol (https://norcca.scrol.net/). The medium was sterilized at 120 ℃ for 15 min. Vitamins, which degrade easily and cannot be autoclaved, were added to the sterilized medium using 0.22 μm syringe filters to ensure sterile conditions before inoculation. The inoculation was performed in a laminar-flow hood using exponentially growing cells under sterile conditions. Cyanocobalamin (Sigma-Aldrich) was used as a B_12_ source in all the experiments. Cultures were maintained at 20 ℃ under a 16:8 h light and dark photoperiod. Light intensity was ~ 150 µmol photons m^− 2^ s^− 1^. Bacterial cells were preserved in glycerol at ˗80 ℃. To start the bacterial culture, a small amount of frozen stock was inoculated into tryptone yeast extract (TY) broth [27] and incubated for 5 days at the same temperature as the alga.

### B_12_ and Nitrogen Experiments

In the first experiment, we studied how varying B_12_ availability affected the growth and fatty acid content in *E. gracilis*. Six different concentrations of B_12_ (0, 20, 60, 90, 120, and 200 ng L^− 1^) were tested, with each treatment consisting of six replicates. The growth of *E. gracilis* was monitored for 6 days by counting cells with a hemocytometer with a chamber depth of 0.1 mm at 20 × magnification with an inverted light microscope (LEICA DMi1). Samples for fatty acid analysis were collected at the end of the experiment by centrifuging the samples at 2500 *× g* for 6 min. A B_12_ concentration of 90 ng L^− 1^ maintained high growth rates and PUFAs levels and was therefore chosen as the sufficient B_12_ concentration for subsequent experiments.

In the second experiment, we investigated the combined effects of B_12_ and nitrogen on growth, as well as the fatty acid and protein content of *E. gracilis*. The starting concentration for algal cells was 10^4^ cells mL^− 1^. The control treatment contained 90 ng L^− 1^ of B_12_ and 131 mg L^− 1^ of nitrogen as ammonium (NH_4_^+^-N). For experimental cultures, the media was modified by limiting nitrogen and B_12_ concentrations to 1/10th of the control levels (0.935 mM and 6.64 pM respectively) to create deficient conditions, keeping all other components of the media identical. To ensure that algal growth was strictly limited by either B_12_ or nitrogen, the algal strain was acclimated to nutrient depleted conditions through two transfers in depleted medium over two weeks. The cells limited to B_12_ and nitrogen were then grown till they reached the exponential growth phase to start the experiment. At the beginning of the experiment, cells were centrifuged at 2500 *× g* for 6 min, washed, and inoculated into the respective treatments. Cells were counted daily (cells mL^− 1^), while fatty acid (µg mg^− 1^ DW) and protein content of total dry biomass were analysed at the end of the experiment (day 6). Cells were stained with 1% Lugol solution and counted using a hemocytometer with a chamber depth of 0.1 mm at 20 × magnification with an inverted light microscope (LEICA DMi1). The specific growth rate (𝜇) was calculated as follows:

1 $$\:{\upmu\:}=\frac{\mathrm{l}\mathrm{n}(\mathrm{N}2/\mathrm{N}1)\:}{\mathrm{t}2-\mathrm{t}1}$$

where N1 and N2 are the cell count at time t1 (initial time point) and t2 (final time point), respectively.

### Algae-Bacteria Co-Culture

In the third experiment, we investigated how different bacteria-to-algae ratios influence growth dynamics, nutrient exchange, and overall interactions between *E. gracilis* and *M. loti*. The bacteria-to-algae ratios were: 1:1, 5:1, 10:1, and 20:1. In the control treatment, *E. gracilis* was grown in culture media supplemented with 90 ng L^− 1^ of B_12_ (cyanocobalamin) without any added bacteria. Algae and bacterial cells were centrifuged at 2500 *× g* for 6 min and 3500 *× g* for 2 min, respectively, washed twice, and then kept in their respective treatments. In co-cultures, both algae and bacteria were batch-cultured in MCW-15 medium, which did not contain B_12_ or added carbon. The starting concentration of algae was 10^4^ cells mL^− 1^, and the bacteria concentration was adjusted to achieve the target ratio (1:1 ratio = 1 × 10^4^, 5:1 ratio = 5 × 10^4^, 10:1 ratio = 1 × 10⁵, and 20:1 ratio = 2 × 10⁵ bacterial cells mL^− 1^). To start the experiment, bacterial cells were counted as individual colony-forming units (CFU mL^− 1^) on solid TY agar plates using the serial dilution method [27]. Each CFU represents the growth of a single viable cell. In this experiment, the algal cells were measured at optical density 680 nm (OD_680_) with a spectrophotometer (Hitachi U-2000) [28]. To validate the measurements of algal cells at (OD_680_) in co-cultures, cells were counted with both hemocytometer and spectrophotometer initially. A strong correlation (*r* = 0.812, *P* < 0.001) was observed between cell counts and OD readings (Supplementary Figure S1). We also monitored bacterial contribution in OD_680_ by measuring the OD_680_ of bacterial culture alone with similar thickness as in our co-culture and the contribution of bacteria was negligible to undetected in OD_680_. Hence, in co-cultures, algal growth measurements were performed using OD_680_.

**Fig. 1:**
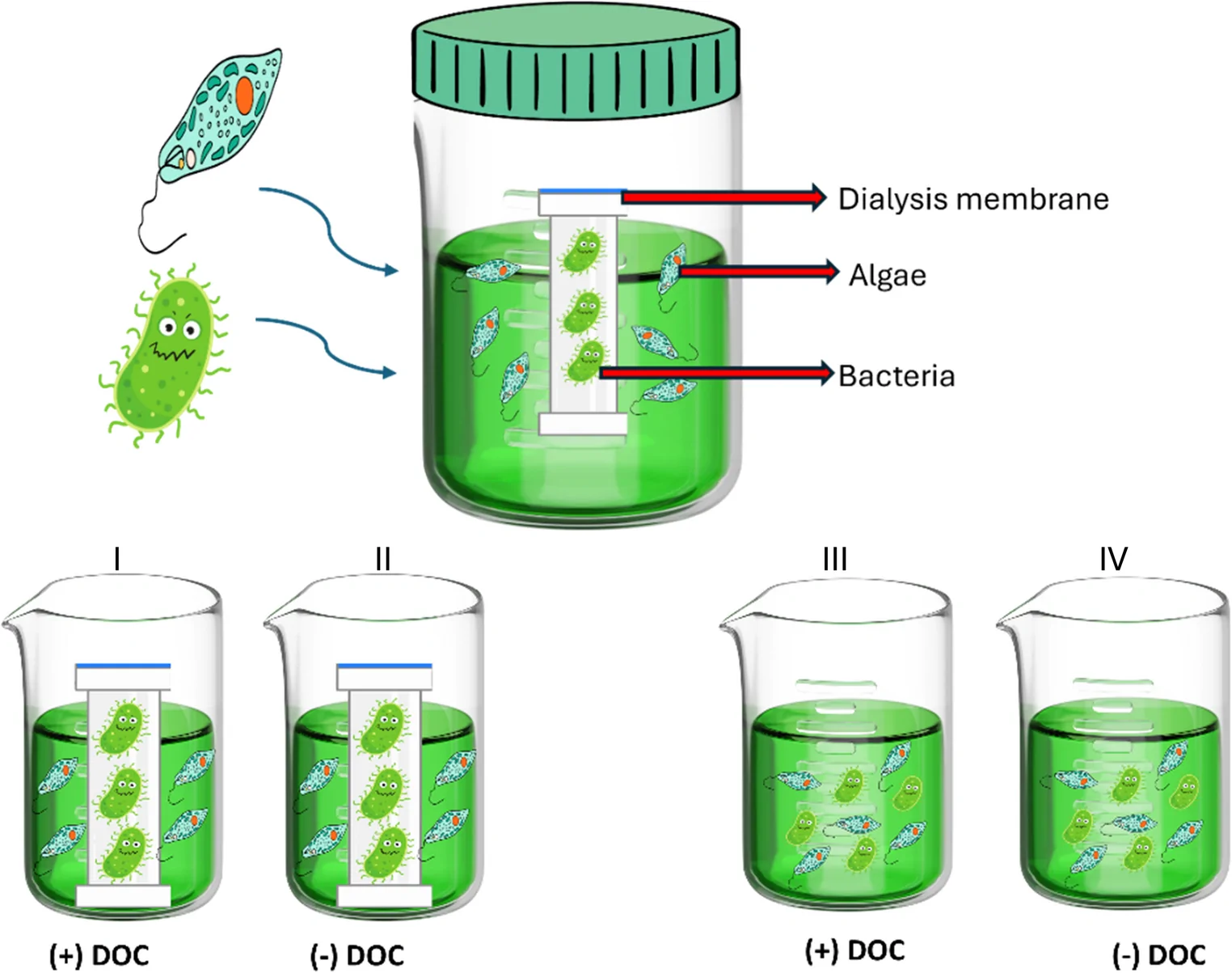
Schematic diagram of experimental setup and treatment design of experiment (IV). The bacterial cells (10^5^ cells L^− 1^) were enclosed in a dialysis membrane and fully immersed in the beaker with algae suspension, allowing the exchange of dissolved chemicals (e.g., nutrients, B_12_, DOC) but preventing the direct interaction of the organisms. A labile-DOC (glycerol, 0.1%) was used as a carbon source. Treatment I: *M. loti* enclosed in a dialysis membrane cultured with *E. gracilis* with the addition of DOC. Treatment II: *M. loti* enclosed in a dialysis membrane cultured with *E. gracilis* without DOC addition. Treatment III: *E. gracilis* and *M. loti* co-cultured with DOC, without a dialysis membrane. Treatment IV: *E. gracilis* and *M. loti* co-cultured without DOC and a dialysis membrane

In the fourth experiment, we investigated algal growth in co-cultures where *E. gracilis* and *M. loti* were physically separated. A dialysis membrane with a 12–14 kDa cutoff was used to create a physical barrier between alga and bacterium, allowing B_12_ molecules to diffuse through. The bacterial cells (10^5^ cells L^− 1^) were enclosed inside the membrane and fully immersed in a beaker with the algae suspension, allowing the exchange of dissolved chemicals (e.g., nutrients, B_12_, DOC) but preventing direct interaction of the organisms (Fig. 1). Glycerol (0.1%) was used as an external labile-DOC source. Glycerol was sterilized at 120 ℃ for 15 min prior to use in the experiment. The experiment consisted of two setups (Fig. 1). In the first setup, algae and bacteria were separated with a dialysis membrane; Treatment I: *M. loti* enclosed in a dialysis membrane co-cultured with *E. gracilis*, with the addition of DOC. Treatment II: *M. loti* inside the dialysis membrane was co-cultured with *E. gracilis* without DOC supplementation. Treatment III: *E. gracilis* and *M. loti* were co-cultured with DOC without membrane. Treatment IV: *E. gracilis* and *M. loti* were co-cultured without DOC supplementation. Algal cell density was evaluated as optical

density at 680 nm at 24 h and then every 12 h for 84 h. Bacterial cells were estimated at 580 nm [29] for treatments without a membrane at 24, 36, 48, 60, and 72 h. Samples were not taken from treatments with a membrane during the experiment to prevent contamination.

### Phagocytosis Test

We aimed to visualize phagocytosis of *M. loti* by *E. gracilis* using fluorescent microscopy and flow cytometry. For this purpose, freshly cultured *M. loti* was collected from the growth medium by centrifugation (5000 *× g* for 1 min), washed once with 1 *×* PBS, and stained in 1 mM DAPI in PBS for 30 min to achieve fluorescent labelling of the bacterial genome. The stained bacteria were washed twice in PBS before adding them to an *E. gracilis* suspension in PBS. After 4 h of co-incubation, the cell suspension was washed 3*×* in PBS before quantification of DAPI fluorescence by flow cytometry using a Beckman Cytoflex (excitation 405 nm, emission filter 450 nm). Algal cells were gated by size, and changes in fluorescence were analyzed using FLOREADA software (https://floreada.io). As a negative control, we attempted to inhibit phagocytosis by the addition of 50 M cytochalasin B but failed to observe any effect. To confirm any increase in fluorescence to be caused by phagocytosis of DAPI-stained bacteria and not by diffusion of dye into algal cells, co-cultured algal cells were also mounted onto microscopy slides and visualized by fluorescent microscopy (Zeiss Axiovert with Axiocam camera and Axiovision software).

### Total Protein Analysis

Total protein content was measured by the modified Lowry method [30]. Approximately 10 mg of freeze-dried algae was homogenized with glass mix beads of 1.1 mm and 0.5 mm in size. The milled biomass was resuspended in 7 mL of 5% (v/v) Triton X-100 and 5% (v/v) glycerol in ethanol solution. The lysate was homogenized by sonicating at 40% amplitude for 15 s, followed by a brief vortex, and 0.5 mL of the lysate sample was collected. Protein was precipitated by adding cold acetone at a ratio of 2:1 (acetone to lysate), vortexed, and kept at -20 °C for 1 h in cold acetone. The pellets obtained after centrifuging at 15,000 *×* g for 10 min at 4 °C were resuspended in 1 mL of 2 M NaOH, and the lysates were stored at 4 °C. Protein was quantified using Bovine serum albumin (BSA Thermo Scientific^®^) standards in 96-well plates. Each sample and standard were pipetted in triplicate, and absorbance was measured at 750 nm using a microplate reader FLUOstar Omega by (BMG). The protein concentration was calculated by comparing the sample absorbance to a standard curve of bovine serum albumin.

Total protein was calculated with the formula as follows:

2 $$\:CVD=\frac{VT/VA}{M}*100$$

where C is the protein concentration (mg L^− 1^) measured by the Lowry assay. VD is the volume of diluent (mL) used to resuspend the protein precipitate. VA is the volume of aliquot (mL) of the lysate used for protein precipitation, VT is the total volume (mL) of lysate, and M is the biomass dry weight (mg) used to assess the protein content.

### Fatty Acid Analysis

Samples were harvested by centrifuging at 2500 *× g* for 6 min. The supernatant was removed, and the algal pellets were frozen at -80 °C and lyophilized. Approximately 2–5 mg of freeze-dried algae was weighed, and lipid was extracted with 1 mL of 2:1 (v/v) chloroform: methanol according to Folch et al. [31]. The tubes were sonicated in ice and water for 10 min. A mild potassium chloride (KCl) solution (0.8%) was added for phase separation, and the lower organic phase was transferred to new tubes. Solvents were evaporated and redissolved in n-hexane. Extracted lipids were transformed into fatty acid methyl esters (FAMEs) with 1% sulfuric acid in methanol at 90 °C for 90 min [32]. Fatty acids were analysed with a gas chromatograph equipped with mass selective detection (Agilent 6890 N GC with Agilent 5973 N MS) using a DB-23 column (60 m 0.25 mm × 0.25 μm). A splitless injection mode was used with helium as a carrier gas at an average velocity of 26 cm s^-1^. The injector temperature was 250 °C. The oven temperature gradient was as follows: initial oven temperature 50 °C for 1 min, then an increase of 15 °C min^-1^ to 150 °C, then 0.50 °C min^-1^ to 170 °C, and finally 2 °C min^-1^ to 230 °C, which was held for 2 min. Quantification of FAMEs was based on mass spectra and the reference standard GLC-538 (Nu-Chek-Prep). The total fatty acid content in algae is shown as micrograms per milligram dry weight (µg mg^-1^ DW), and the fatty acid composition as weight% (w%).

### B_12_ Analysis

B_12_ extraction methods were adapted from Watanabe et al. [33] and Kumudha [34] with minor modifications. Specifically, ethanol was replaced with methanol as the extraction solvent, and the extraction temperature was reduced to 60 ℃. Extraction efficiency was evaluated using 80% ethanol and 80%, 90%, and 100% methanol; 90% methanol produced the highest yield. Among the tested incubation temperatures (60, 70, 80, 90, and 98 ℃), incubation at 60 ℃ for 20 min resulted in the optimal yield. Prior to extraction, methanol was degassed with nitrogen gas (N₂) for 30 min to remove dissolved oxygen. We used a porous sparger to achieve small bubbles for effective O_2_ removal. B_12_ from alga and bacterium was extracted using identical protocol from freeze-dried biomass and pelleted cells respectively. For bacteria, 100 mL of culture (containing 6.3 × 10^7^ cells mL^− 1^) was centrifuged and pelleted, washed 3 times with PBS buffer (centrifuging 5,000 *× g* for 10 min). Approximately 5–10 mg of freeze-dried algal biomass and pelleted bacterial cells were suspended in 2 mL of degassed 90% methanol, briefly flushed, and capped under a nitrogen stream to replace the airspace with nitrogen. Samples were incubated at 60 °C for 20 min, after which samples were centrifuged at 4,000 *× g* for 5 min, and the supernatant was transferred to a 5 mL Eppendorf tube [34, 35]. The supernatant was evaporated to near-dryness using a vacuum centrifuge concentrator at 45 °C and to dryness under N_2_ flow. The residue was redissolved in 380 µl of solvent A (20 mM ammonium formate in water with 0.1% formic acid), and the mixture was centrifuged at 10,000 *× g* for 5 min. The supernatant was transferred to an LC vial and added with 20 ng of ^13^C and ^15^N enriched riboflavin (dioxopyrimidine-^13^C_4_, ^15^N_2_; isotopic enrichment ≥ 98.0%; Sigma Aldrich 705292), also known as vitamin B_2_, as an internal standard. The concentration of B_12_ was then analysed with a liquid chromatograph (Agilent 1290 Infinity) equipped with a quadrupole time-of-flight mass selective detector (Agilent Technologies 6540 Q-TOF). A Waters Acquity HSS Cyano column (1.8 μm, 2.1 × 100 mm) was used [36]. The mobile phase consisted of (solvent A) 20 mM ammonium formate with 0.1% formic acid, and (solvent B) acetonitrile (ACN), for a run time of 12 min. All solvents and chemicals were LC-MS grade. The LC gradient was 0 min: 2% B; 7 min: 30% B; 9 min: 90% B; 12 min to the end: 2% B. Injection volume was 40 µl and flow rate was 0.5 mL min^− 1^. The analysis was performed with a quadrupole time-of-flight mass selective detector (Agilent Technologies 6540 Q-TOF) with an Assisted Jet Stream (AJS) ion source. MS conditions, Collision condition (CE), and retention times (RT) for each analyte and internal standard (IS) are given in Table 1.

**Table 1:** HPLC-MS/MS conditions used to monitor different forms of cobalamin and vitamin B_2_

Compound	Transitions monitored	CE (V)	RT (min)
Vitamin B_2_	383.15 → 249.09	28	3.887
OH-Cbl	664.79 → 147.09	55	4.127
Ado-Cbl	790.33 → 147.09	61	5.522
Me-Cbl	672.88 → 147.09	43	6.481
CN-Cbl	678.29 → 147.09	41	4.706

Note: CE is the collision energy and RT is the retention time. Vitamin B_2_ (riboflavin) acts as the internal standard to test the stability of the HPLC-MS. It is ^13^C- and ^15^N-enriched vitamin B_2_ (dioxopyrimidine-^13^C_4_, ^15^N_2_; isotopic enrichment ≥ 98.0%) (Sigma Aldrich 705292). OH-Cbl, hydroxocobalamin; Ado-Cbl, adenosylcobalamin; CN-Cbl, cyanocobalamin; Me-Cbl, methylcobalamin

Cobalamin (B_12_) has primarily four forms: adenosylcobalamin (Ado-Cbl), cyanocobalamin (CN-Cbl), methylcobalamin (Me-Cbl), and hydroxocobalamin (OH-Cbl). The biologically active forms are Ado-Cbl and Me-Cbl [19]. Both Ado-Cbl and Me-Cbl are highly light-sensitive and rapidly degrade to OH-Cbl when exposed to light [37]. Although all extraction steps were conducted under dim red light to minimize conversion of bioactive forms to OH-Cbl, OH-Cbl remained the dominant form detected, while other forms were below detection limits. Due to the photolability of Ado-Cbl and Me-Cbl and the limitations of the extraction protocol, the reported OH-Cbl concentrations should be considered a conservative estimate of the total cellular B₁₂ pool.

### Statistical Analysis

We used Levene’s test to examine the uniformity of variances and the one-sample Kolmogorov-Smirnov test to assess normality, with a significance level set at alpha < 0.05. Results showed that the cell count, protein, and total fatty acid content satisfied the assumptions of normality and homogeneity of variances. We used Permutational Analysis of Multivariate Dispersion (PERMDISP) to assess whether there were notable differences in the dispersion of fatty acid composition among samples. Fatty acids (w%) data were arcsine square root transformed prior to the analysis [38]. The results for all tested experiments (dose-response, nutrient starvation and algal bacterial co-culture) showed that there were no significant differences in dispersion (permutation: free, number of permutations:999, distance: Bray-Curtis, *p* > 0.05), which permitted us to advance with a Permutational Multivariate Analysis of Variance (PERMANOVA) for assessing fatty acid profiles between the treatments [39]. We employed non-metric multidimensional scaling (NMDS) to illustrate the variations in fatty acid (FA) composition of *E. gracilis* across different treatments. Bray-Curtis similarity was used to construct the similarity matrix. To evaluate the fit of the NMDS ordinations, we considered stress values below 0.2 to indicate a good fit to the data [40]. The one-way ANOVA and familywise Tukey test were used to test for significant differences between treatments at the 95% confidence level. All analyses were performed in R 4.1.1 (2021-08-10) or Primer 6 with Permanova + add on [39].

## Results

### Effects of B_12_ and Nitrogen on Growth and Nutritional Quality of *Euglena*

**Fig. 2:**
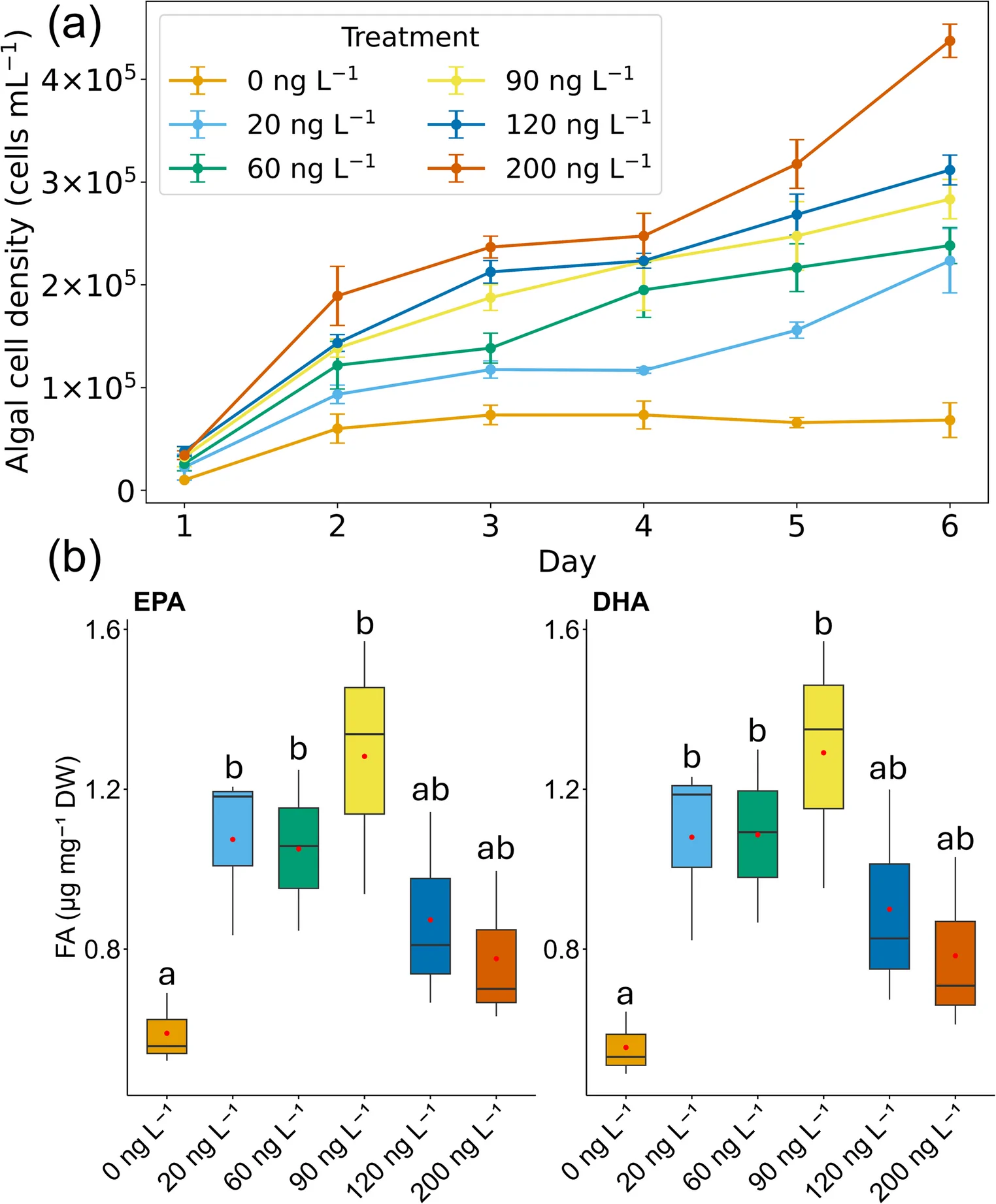
B_12_ dose-experiment in culturing *E. gracilis.* (**a**) *E. gracilis* cell densities at different levels of B_12_ concentration (0, 20, 60, 90, 120, and 200 ng L^− 1^). (**b**) EPA and DHA mass fractions in *E. gracilis* with varying levels of B_12_ (0, 20, 60, 90, 120, and 200 ng L^− 1^). Boxes indicate the interquartile range (IQR; Q1–Q3), with the median shown as a horizontal line. Whiskers extend to the most extreme values within 1.5×IQR. Each treatment has 6 replicates. * and ** denote the significance level at *p* < 0.1 and *p* < 0.05, respectively

A significant growth response of *E. gracilis* was observed with increasing availability of B_12_ (Fig. 2a). By day 6, algal growth was enhanced by 6-fold compared to the treatment without B_12_ (0 ng L^− 1^). The highest dose of B_12_ (200 ng L^− 1^) promoted the fastest growth rate (𝜇) of alga. Cultures without B_12_ stopped growing after two days (Fig. 2a), with cell counts significantly lower than those in other treatments (ANOVA, *p* < 0.01) (Supplementary Table S1). Even the highest concentration of B_12_ (i.e., 200 ng L^−1^) did not reach a saturation point (Supplementary Figure S2). The availability of B_12_ had a tendency of unimodal effect on EPA and DHA content of the algae (Fig. 2b) as well as an effect on the total fatty acid content (Supplementary Figure S3). However, the pattern was not significant and only 0 ng L^−1^ differed from other treatments (20 ng L^−1^, 60 ng L^−1^, and 90 ng L^−1^) of B_12_. The mass fractions of EPA and DHA and total fatty acid content increased with B_12_ concentrations up to 90 ng L^−1^; however, both declined at B_12_ concentrations exceeding this threshold. Fatty acid profiles also differed among different levels of B_12_ (Supplementary Figure S4). B_12_ concentration in the culture media had a significant effect on the FA w% (PERMANOVA, pseudo-F _5, 12_ = 3.21, *p* = 0.014) (Supplementary Table S2). Fatty acids such as 20:0, 16:1n-6, 16:2n-7, 16:3n-6, 18:2n-6, 22:6n-3 increased with increasing B_12_ concentration, whereas 16:0, 18:0, 18:3n-3 decreased (Supplementary Table S3). The most abundant fatty acids in *E. gracilis* were 18:3n-3, 16:0, and 16:4n-3, which together accounted for 45–51% of all fatty acids content. Total fatty acid content ranged from 22 to 109 µg mg^−1^ DW, and EPA and DHA ranged from 1.8 to 9.1 and 1.4–8.7 µg mg^−1^ DW, respectively.

**Fig. 3:**
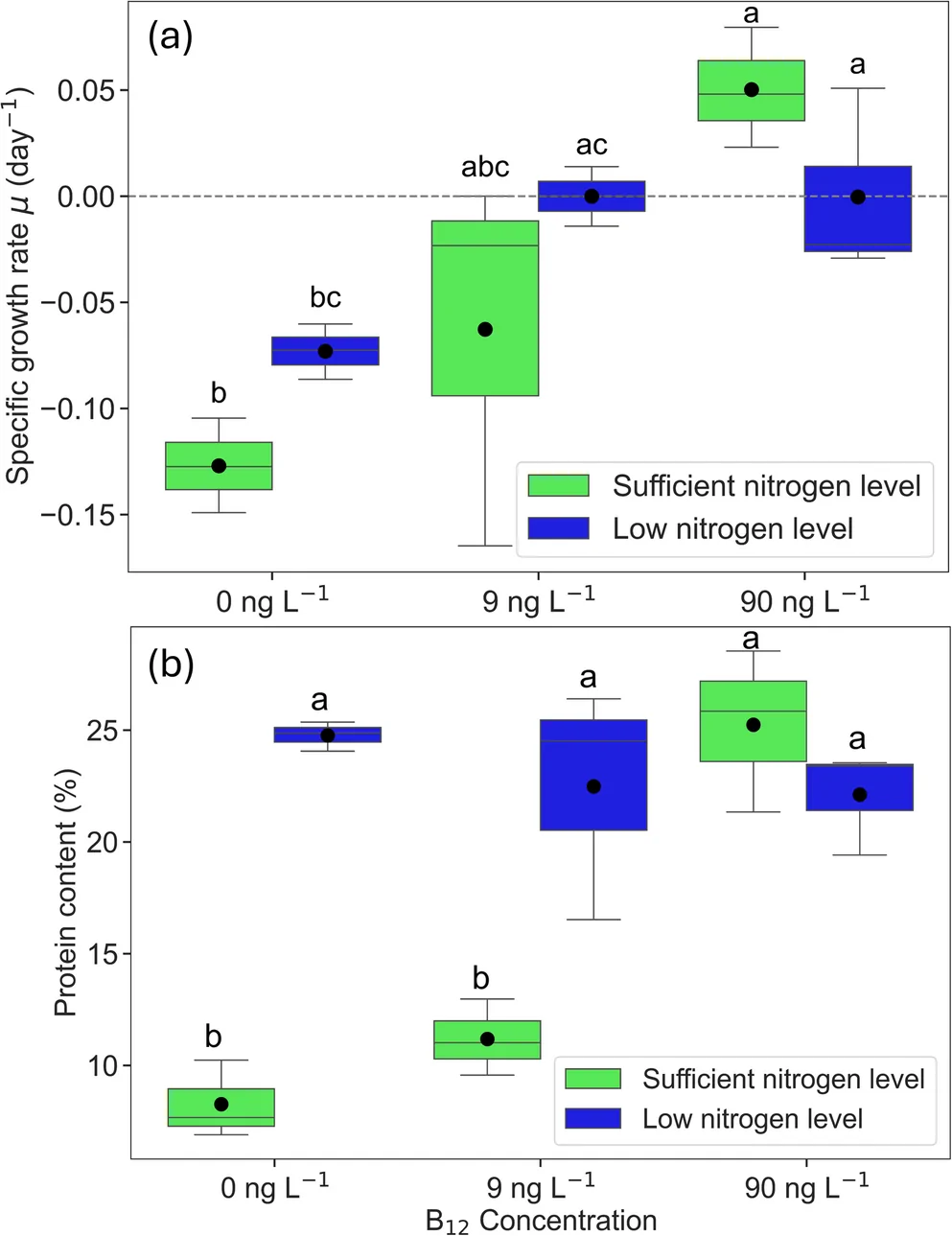
Interaction of nitrogen and B_12_ availability on the (**a**) growth rate and (**b**) protein content (%) of *E. gracilis*. (**a**) B_12_ deprivation (0 ng L^-1^) affected negatively the growth of *E. gracilis* in both low and sufficient nitrogen conditions. (**b**) B_12_ limitation (≤ 9 ng L^-1^) decreased cellular protein content in *E. gracilis* in sufficient nitrogen conditions but not in low nitrogen conditions. Boxes indicate the interquartile range (IQR; Q1–Q3), with the median shown as a horizontal line. Whiskers extend to the most extreme values within 1.5×IQR. Green boxes represent a sufficient nitrogen level, and blue boxes represent a low nitrogen level. The letters above the box plots indicate significant differences between treatments; treatments marked with different letters are statistically distinct from each other

To evaluate the combined effects of nitrogen and B_12_ availability, we conducted a 2 × 3 factorial experiment, with two levels of nitrogen (sufficient and low) and three levels of B_12_ (0, 9, and 90 ng L^− 1^). The specific growth rate was significantly lower in B_12_-deplete conditions, irrespective of nitrogen availability, compared to all other treatments (Fig. 3a). Under sufficient nitrogen condition, growth rate dropped from (µ = 0.05 day^− 1^) in control with 90 ng L^− 1^ of B_12_, to (µ = -0.06 day^− 1^) in 9 ng L^− 1^ B_12_, and further to (µ = -0.13 day^− 1^) in the treatment without any B_12_. No net growth (µ = 0 day^− 1^) was observed under low nitrogen levels at 90 ng L^− 1^ and 9 ng L^− 1^ of B_12_. A significant interaction between nitrogen and B_12_ availability was observed in the algal total protein content. B_12_ limitation (≤ 9 ng L^− 1^) decreased cellular protein content in *E. gracilis* in sufficient nitrogen conditions but not in low nitrogen conditions (Fig. 3b). Protein content was 3-fold higher in the treatment with B_12_ than without B_12_. The control (sufficient nitrogen with 90 ng L^− 1^ of B_12_) showed the highest protein content of 25% of dried biomass, but protein content significantly dropped to around 12% when B_12_ was depleted, and to 7% when there was no B_12_ under sufficient nitrogen condition. Although under low nitrogen conditions, no significant difference in protein content was observed among B_12_ levels, protein content tends to be slightly higher in lower B_12_ levels. Varying availability of B_12_ and nitrogen had no significant effect on the fatty acid profiles of *E. gracilis* (Supplementary Figure S5). α-Linolenic acid (18:3n-3) was the most abundant across all treatments (Supplementary Table S4). Other abundant fatty acids were palmitic acid (16:0) and EPA (20:5n-3). EPA content was highest with a mean of 12.3% in low nitrogen conditions with 90 ng L^− 1^ of B_12_.

### Alga-Bacterium Interaction

**Fig. 4:**
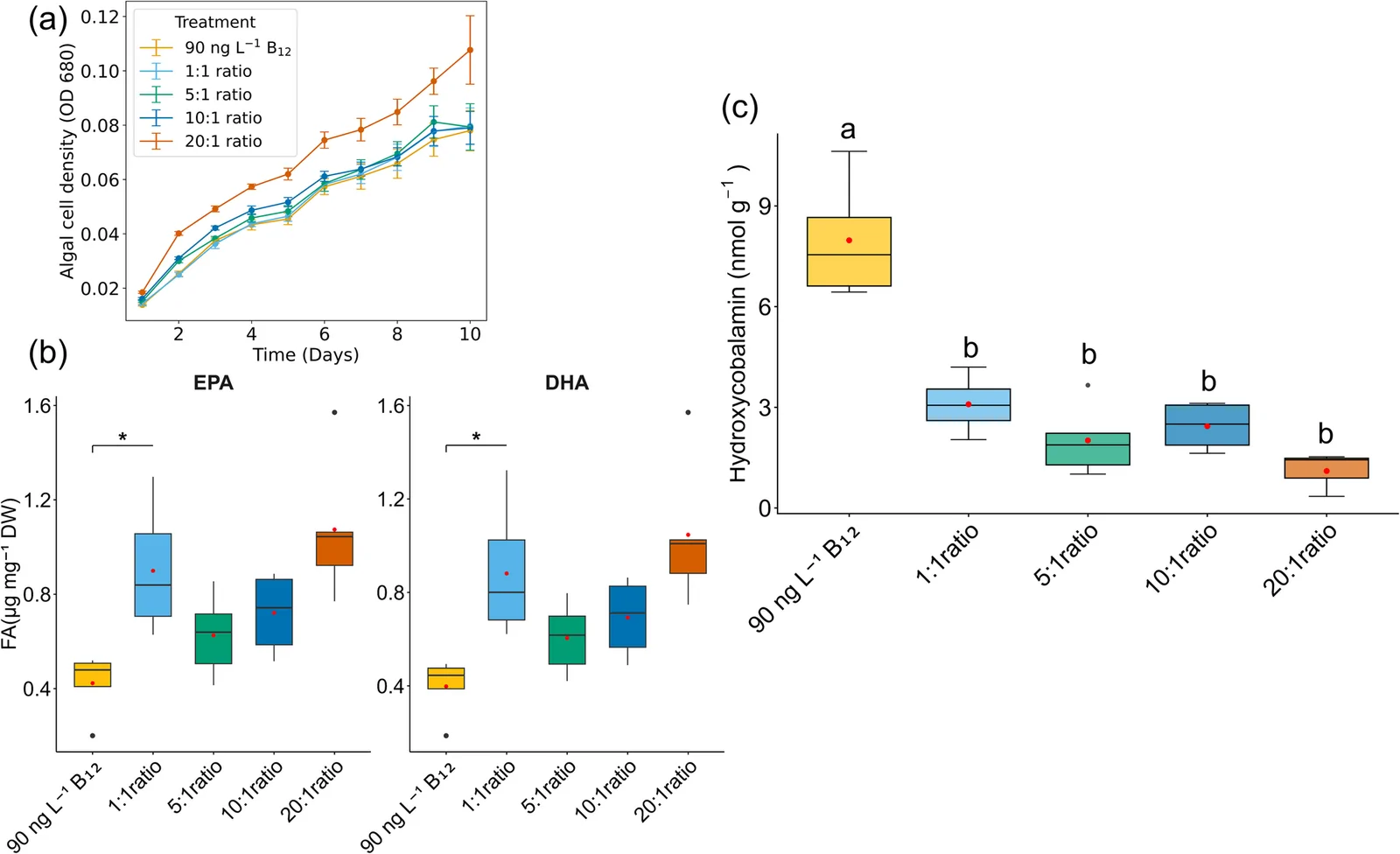
Effects of the amount of bacteria on the growth, the PUFA content, and B_12_ content of *E. gracilis*. (**a**) Cell density of *E. gracilis* at different bacteria-to-algae ratio. Yellow colour with 90 ng L^− 1^ B_12_ represents the control that is grown with exogenous B_12_ without adding bacteria, all the other colours denote different bacteria-to-algae ratios. Each treatment contains 6 replicates. (**b**) EPA and DHA mass fractions (µg mg^− 1^ DW) on day 10. * denotes significance level at (*p* < 0.1). (c) Concentration of hydroxy cobalamin (OH-Cbl; nmol g^− 1^) in *E. gracilis* at different bacteria-to-algae ratios with 5 replicates per treatment. Boxes indicate the interquartile range (Q1–Q3), with the median shown as a horizontal line. Whiskers extend to the most extreme values within 1.5×IQR, and points beyond the whiskers represent outliers. The letters above the box plots indicate significant differences between treatments; treatments marked with different letters are statistically distinct from each other (*p* < 0.05)

Alga grew better in co-cultures with *M. loti* than in the control treatment, i.e., culture with 90 ng L^− 1^ of B_12_ (Fig. 4a). The highest growth was observed at the 20:1 bacteria-to-algae ratio, resulting in a 1.4-fold increase compared to cultures with 90 ng L^− 1^ of B_12_. Algal growth between the bacteria-to-algae ratio 20:1 and the control differed on all days except day 9 and day 10 (Supplementary Table S5).

Co-culturing *E. gracilis* with *M. loti* at different ratios significantly influenced the EPA and DHA levels in the algae (Fig. 4b). EPA and DHA levels were significantly higher (independent t-test, *p* < 0.05) only in the co-culture with a 1:1 bacteria-to-algae ratio compared to the control treatment. The algal fatty acid profiles differed between the bacteria-to-algae treatments (PERMANOVA, pseudo-F _4,23_ = 1.816, *p* = 0.012) (Supplementary Figure S6, Supplementary Table S6). A significant difference in mean total fatty acids among treatments (ANOVA, *p* < 0.001) was observed (Supplementary Figure S7) (Supplementary Table S6). EPA was more abundant in *E. gracilis* in co-cultures with a 1:1 bacteria-to-algae ratio, while saturated fatty acids (16:0, 18:0, 20:0) were more prevalent in the control treatment with 90 ng L⁻¹ of B_12_ (Supplementary Table S7).

The level of cellular B_12_ as OH-Cbl mass fractions in *E. gracilis* was more than two-fold in the culture with 90 ng L^− 1^ of B_12_ as compared to the co-cultures with higher bacteria-to-algae ratios (Fig. 4c). There was no correlation between the specific growth rate and the cellular B_12_ content of *E. gracilis* (Supplementary Figure S8). No significant differences in OH-Cbl concentrations were observed among the different bacterial-to-algal ratios. CN-Cbl measurements in all samples were below the detection limit. The bacterium *M. loti* produced B_12_ predominantly in the form of Me-Cbl (61.1%) and Ado-cbl (27.5%) (Supplementary Figure S9). However, Me-Cbl and Ado-Cbl were detected in our samples but not quantified due to low concentrations (peak signal-to-noise ratio mostly below 3:1).

**Fig. 5:**
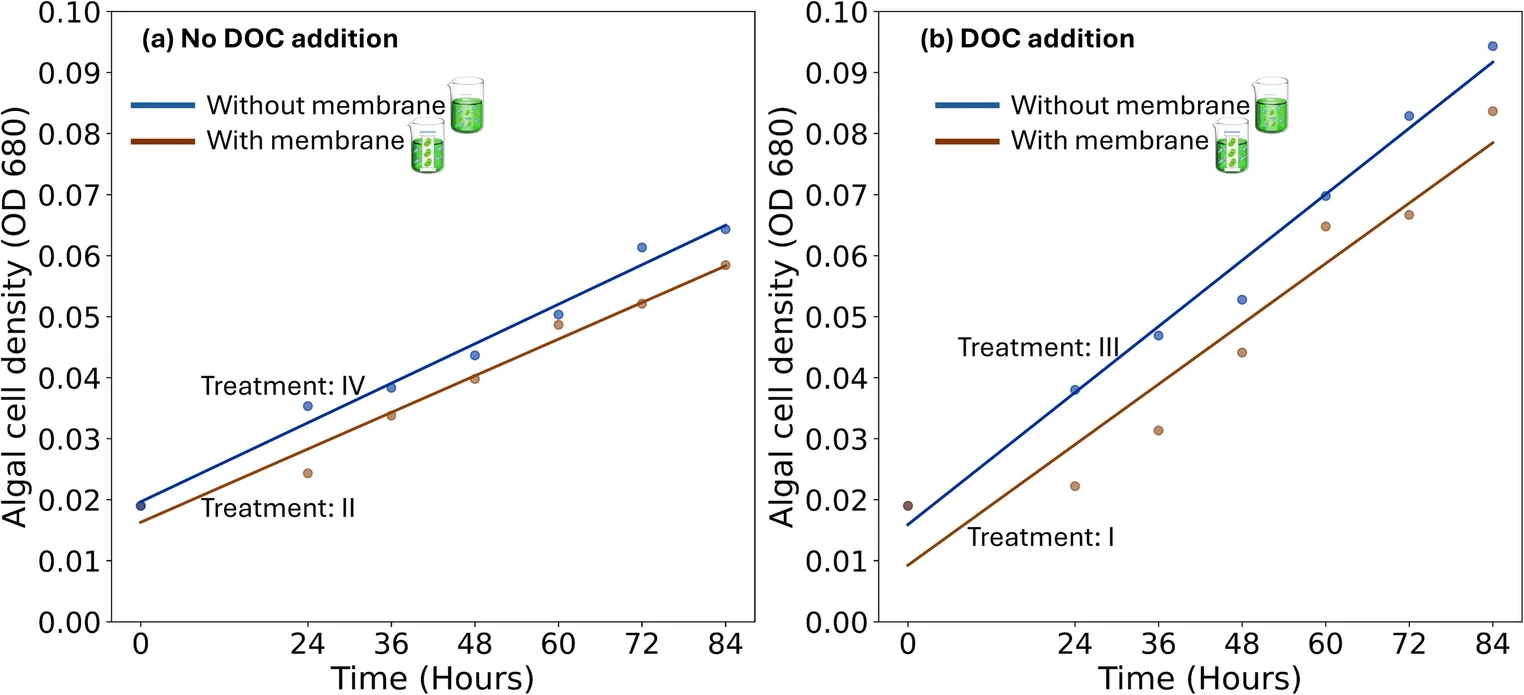
Effects of dialysis membrane and a labile-DOC (glycerol, 0.1%) addition on algal cell growth rate. (**a**) Growth of *E. gracilis* without DOC addition, and (**b**) growth of *E. gracilis* with DOC addition. Each treatment has three replicates (*n* = 3). Blue and brown lines denote the treatment without and with a dialysis membrane, respectively. DOC supplementation significantly enhanced algal growth. No significant difference in algal growth was observed between treatments with or without a membrane within 84 h of culture. This indicates that physical separation of alga and bacterium had no measurable effect on the growth of *E. gracilis*

The presence of labile-DOC (glycerol, 0.1%) significantly enhanced algal growth across all the treatments (Fig. 5). The OLS model explained 94.1% of the variance in algal cell density (adjusted R² = 0.934, F_7, 64_ = 145.20, *p* < 0.001) (Supplementary Table S8). The interaction between time and treatment was significant (F_3, 64_ = 22.82, *p* < 0.001), indicating that growth rates (slopes) differed among all treatments. Estimated slopes were 0.0010 OD hour-1 for treatment III (*E. gracilis + M. loti* without membrane and with DOC) and treatment I (*E. gracilis + M. loti* in membrane with DOC), 0.0006 OD hour-1 for treatment II (*E. gracilis + M. loti* in membrane without DOC), and 0.0005 OD hour-1 for treatment IV (*E. gracilis + M. loti* without membrane without DOC), with the interaction accounting for an additional 14.3% of variance (ΔR² = 0.143). The physical separation of algae and bacteria did not significantly affect algal growth rate, likely due to high variability among treatments (Fig. 5, Supplementary Table S8). Furthermore, when the membrane was absent, *M. loti* exhibited a significantly higher growth rate in the presence of DOC, as compared to the treatment without DOC (Supplementary Figure S10).

While an increase in fluorescence of algal cells co-cultivated with fluorescent bacteria further supports our hypothesis of phagocytosis (Supplementary Figure S11), the possibility of this being an artifact caused by dye diffusion rather than phagocytosis remains. Any attempts to verify the presence of bacteria within algal cells using fluorescence microscopy were inconclusive, as the strong autofluorescence of chloroplasts masked the signal, even with short-wavelength dyes.

## Discussion

This study confirmed that *Euglena gracilis* is a B_12_-auxotroph, consistent with previous findings [10, 41, 42], and improved our understanding of the interactive effects of B_12_ and nitrogen availability on the nutritional quality of *E. gracilis*. We showed that B_12_ availability alters EPA and DHA mass fractions in *E. gracilis*. This could have negative effects on aquatic ecosystem as these essential FAs play a critical role in the nutritional physiology of aquatic organisms including zooplankton and fish [43, 44]. For instance, a reduction in EPA and DHA at primary producer level is directly associated to decreased growth and reproduction in keystone zooplankton taxa such as Daphnia [45–47]. Fish fed marine larvae rich in EPA and DHA have enhanced growth [48]. Furthermore, changes at the lower trophic levels may cascade throughout the food web and alter EPA and DHA levels in fish and thus affect human dietary intake [49, 50]. Co-culturing *E. gracilis* with a B_12_-synthesizing bacterium enhanced algal growth more than B_12_ supplementation alone. Enhanced algal growth in co-cultures corresponded with lower cellular levels of B_12_, compared to algae grown with B_12_ supplementation. These findings are suggestive that *E. gracilis* may obtain additional growth-promoting nutrients, beyond B_12_, from *M. loti*, thereby further enhancing the algal growth. Algal-bacterial symbioses studies have shown that bacteria affects algal growth and metabolism by influencing amino acid biosynthesis, production of signaling molecules, supply of iron-chelating compounds, and other growth promoting factors beyond just vitamin supply [51–53]. These results also suggest the occurrence of luxury uptake in *E. gracilis*. Similar to the effect of B_12_ enrichment, DOC supplementation significantly enhanced algal growth, as expected for a mixotrophic algae. Physically separating algal and bacterial cells with a dialysis membrane, while still permitting the exchange of B_12_ and other small molecules, did not significantly affect algal growth. Our results improve understanding of how B_12_ availability influences *E. gracilis* growth and the accumulation of biochemical compounds accumulation across different scenarios.

B_12_ supplementation significantly increased the growth of *E. gracilis* throughout the range of B_12_ concentrations tested, and even the highest dose of B_12_ (200 ng L^− 1^) did not establish a growth saturation point (Supplementary Figure S2). In laboratory cultures, a minimum of 20–50 ng L^− 1^ cobalamin is required to maintain algal growth in axenic conditions [2, 54]. In nature, the concentrations of vitamin B_12_ are extremely low, ranging from 0 to 3 pM (0–4 ng L^− 1^) in seawater and approximately 10 pM (~ 14 ng L^− 1^) in freshwater [55–57]. Half-saturation constants (Ks) for maximum growth have varied among algal species, with the dinoflagellate *K. mikimotoi* requiring 13.1 pM (17.8 ng L^− 1^) and *Aureococcus anophagefferens* requiring 3.49 pM (4.73 ng L^− 1^) of B_12_ [58]. Thus, B_12_ is likely one of the key factors limiting microalgal growth and is a key factor in regulating the biomass of B_12_ auxotrophic algae across freshwater and marine ecosystems [10, 59, 60]. The EPA and DHA mass fractions in *E. gracilis* responded to increasing B_12_ availability in a unimodal pattern. EPA and DHA mass fractions initially increased with increasing vitamin B_12_ concentrations up to 90 ng L^− 1^ but declined at higher concentrations (Fig. 2b). B_12_ acts as a cofactor for a methionine synthase which is essential for DNA, lipids, and amino acid synthesis. At low B_12_ availability, which coincided with slow algal growth, methionine production may be compromised, leading to cellular alterations, such as folate trapping and disrupted C1 [9, 61]. Cell division is constrained as protein and nucleic acid synthesis slows down due to B_12_ limitation, thus the cell redirects photosynthesized carbon from protein synthesis to the synthesis of storage compounds, such as lipids. This mechanism is analogous to the well-established effects of phosphorus and nitrogen limitation on algal growth and lipid content [62, 63]. *Phaeodactylum tricornutum*, a unicellular diatom, accumulates 20–30% of its dry cell biomass as lipids in low nitrogen conditions [64]. A similar mechanism has been suggested in iron fertilization experiments. Elevated iron availability supports faster growth, but lipid accumulation is highest when iron availability is limited [65]. Nutrient limitation has been widely used to increase lipid content in microalgae [66, 67]. At the highest B_12_ concentration, the low EPA and DHA levels are most likely due to growth-dilution, consistent with the rapid growth observed at this concentration. The decline in polyunsaturated fatty acids (PUFAs) at high B_12_ concentrations might represent a metabolic adaptation that prioritizes energy utilization over lipid accumulation [68].Vitamin B₁₂ acts as a cofactor for methylmalonyl-CoA mutase (MCM), that processes propionate-derived carbons into tricarboxylic acid (TCA) cycle via succinyl-CoA that fuels cell division [69]. With the increase in cell growth, the carbon may be directed towards membrane biogenesis, and synthesis of saturated fatty acids that involves few enzymatic steps than metabolically costly PUFAs synthesis pathways [69–71]. Reduced EPA and DHA production at the primary producer level may decrease the trophic transfer of these molecules, which may ultimately decrease EPA and DHA levels in fish and potentially dietary intake for humans [49, 50]. Eutrophic freshwaters may be particularly sensitive to decreased EPA and DHA levels [49, 72]. In humans EPA and DHA are vital for cardiovascular health, cell membranes, and cellular signalling [73–76].

The lower level of cellular B_12_ [as Hydroxo-cobalamin (OH-Cbl)] in *E. gracilis* maintained in co-cultures indicate that the alga may allocated most of the available B_12_ for growth, resulting in reduced cellular B_12_ levels. In contrast, the higher cellular B_12_ concentrations observed in cultures without bacterium suggests that the alga may engage in luxury consumption, i.e., uptake of non-limiting nutrients beyond immediate growth needs [77]. Thus, algal cultures supplemented with commercial B_12_ may have been limited by nutrients other than B_12_. *E. gracilis* is a mixotroph, and bacteria are also known to supply algae with other molecules and vitamins, such as iron, through the production of photosensitive siderophores [78]. In the co-culture experiment, *M. loti* enhanced the growth of *E. gracilis* much more than B_12_ supplementation alone (Fig. 4a). This is consistent with previous studies that showed algae and bacteria may form a mutualistic relationship, with bacteria releasing inorganic nutrients and vitamins to enhance the growth of algae and algae providing photosynthate to the bacteria [78, 79]. It is important to note that, while our cultures were routinely monitored for contamination using microscopy and agar plating, these methods do not confirm the cultures were axenic, unlike 16s rRNA gene sequencing or epifluorescence microscopy with nucleic acid staining.

In the current study, the samples for B_12_ analysis were collected at the end of the experiment, thus changes in cellular B_12_ levels over time could not be resolved. More frequent monitoring of cellular B_12_ is needed to characterize B_12_ dynamics in algae. B_12_ consists of several variants and in seawater Me-Cbl was the dominant variant of B_12_ accounting for more than 80% of total cobalamin [80]. Although Me-Cbl and Ado-Cbl were detected in our study, the concentrations were below reliable quantification. While we took great care not to expose the samples to light, it is possible that Ado-Cbl and Me-Cbl were converted into OH-Cbl [37]. Alternatively, lyophilization or extraction may have promoted the conversion of Ado-Cbl and Me-Cbl to OH-Cbl. In *E. gracilis* cells, OH-Cbl also acts as reservoir of cobalamins [81].

*E. gracilis* grew best when labile-DOC was added, which is consistent with previous studies showing *E. gracilis* attains higher biomass when supplemented with dissolved organic carbon [82, 83]. *E. gracilis* can metabolize a wide range of carbon sources, which provides energy to support faster cell division and higher biomass production [82]. These findings align with previous studies showing that mixotrophs combine autotrophy and heterotrophy to acquire carbon and other nutrients, such as N and P [84]. The ability to switch between autotrophic and heterotrophic modes provides mixotrophs with a competitive advantage over autotrophs, particularly in high-nutrient and high algal production, when algal DOC leakage may be high. Heterotrophic bacteria are important in ecosystem functioning, specifically in the decomposition of organic matter and nutrient cycling [85, 86]. Although our study did not unequivocally confirm that phagocytosis of B_12_-producing bacteria in *E. gracilis*, the observed slight increase in fluorescence of algal cells co-cultivated with fluorescent bacteria suggests that phagocytosis may support carbon uptake and B_12_ supply for this alga (Supplementary Figure S10). It should be noted that possible dye diffusion and the failure of cytochalasin B to inhibit uptake cannot be neglected. Hence, the flow cytometry data (Supplementary Figure S11) are suggestive but not conclusive. A transcriptomics study showed that a phagotrophy-related gene was upregulated along an increasing DOC gradient in a mixotrophic algal species (*Cryptomonas sp.*) [87]. Notably, phagocytosis has been previously documented in *E. gracilis* [88]. Phagocytosis may become increasingly relevant under B_12_-depleted conditions.

The results from the B_12_ and nitrogen experiments indicate strong metabolic co-dependency (Fig. 3). In a B_12_-deplete environment, a B_12_ auxotrophic alga cannot utilize nitrogen effectively (Fig. 3b). The synergistic effects of B_12_ and nitrogen on algal biochemistry and growth have important implications for nutrient cycling and primary production in aquatic ecosystems. Nitrogen is essential for the biosynthesis of amino acids, nucleotides, and other key biomolecules; hence, nitrogen deprivation can lead to significant changes in both photosynthetic activity [89] and biochemical composition, such as protein, lipids, and carbohydrate synthesis in algae [90, 91]. Under sufficient nitrogen conditions, both growth and protein contents were reduced under B_12_-depleted conditions, which highlights the importance of B_12_ in essential metabolic processes. The lower specific growth rate and protein content in *Euglena* in B_12_-deprived conditions are likely due to limited methionine synthesis, which leads to reduced efficiency in nitrogen assimilation and protein synthesis, and hence growth [8, 92]. For example, nitrate addition alone enhanced diatom growth, even though 60% of them were B_12_ auxotrophs. However, when both B_12_ and nitrogen were added, the phytoplankton community shifted to dinoflagellate-dominance (90% of taxa being B_12_ auxotrophic). This suggests that the availability of B_12_ affects nitrogen metabolism in algae [58, 93].

Under low-nitrogen conditions, *E. gracilis* protein content remained high even when B_12_ availability was low. Carell [94] reported that this phenomenon is due to cell gigantism, where protein content increases in B_12_ deprived conditions. Under B_12_ and nitrogen deprivation, cell division is hindered due to impaired DNA synthesis, but chloroplast growth continues independently. The combined addition of B_12_ and nitrogen can led to enhanced chlorophyll production in eukaryotic algae [95]. Chloroplasts are rich in proteins and cells accumulate more and larger chloroplasts while being unable to divide. This produces “giant” cells with high protein content per cell. A similar observation was made by Bunbury et al. [9], where the loss of cell viability due to B_12_ deficiency was less severe when cells were also limited for nitrogen. When both B_12_ and nitrogen are limited, the algae produce lower levels of reactive oxygen species (ROS), which help to protect cells and minimize cell death, enhancing algal survival [9]. However, it should be noted that our measurements only represent total protein content rather than cellular quotas, which cannot distinguish between increased protein synthesis at cellular level and arrested cell division coupled with accumulation of protein-rich chloroplast [96–98]. Further investigations on quantification of protein per cell would provide mechanistic clarity of this work.

## Conclusion

This study shows the complex interplay between vitamin B_12_ availability and nutrient conditions, and how these cause shifts in algal physiology and biochemistry. The results revealed that B_12_ limitation reduces nutritional quality, such as fatty acids (EPA, DHA) and protein content, which may reduce production at the upper trophic levels. Thus, the microbial interaction should be further explored and linked to the food web and to the functioning of aquatic ecosystems. Microbial interactions play a crucial role in meeting the B_12_ requirement in algae. Any alteration in this interaction leads to changes in algal physiology and biochemistry. Our study suggests that mixotrophs can tap into microbial loop utilizing bacteria as both a source of B_12_ and organic matter, that gives them a distinct competitive edge in aquatic ecosystem. 

## Supplementary Information

Below is the link to the electronic supplementary material.

Supplementary Material 1 (DOCX 55.8 KB)

Supplementary Material 2 (DOCX 2.05 MB)

## Data Availability

The data that supports the findings of this study are available in the Supporting Information of this article.
