# Supplementary material for: Vitamin B12 Availability and Interaction with Bacterium Mesorhizobium loti Affect Growth and Biochemical Composition of Mixotrophic Alga Euglena Gracilis
Source: Microb Ecol. 2026 Jun 23;89:169. doi: 10.1007/s00248-026-02816-0 (PMC13542019; doi:10.1007/s00248-026-02816-0)
Supplement: Supplementary file 1 [file 248_2026_2816_MOESM1_ESM.docx]

**Table S1:** Tukey familywise significance test between treatments without B_12_ (0 ng L^-1^) and with varying levels of B_12_ (20 ng L^-1^, 60 ng L^-1^, 90 ng L^-1^, 120 ng L^-1^, and 200 ng L^-1^) in 6 days.

| Day | **group1** | **group2** | **meandiff** | **p-adj** | **lower** | **upper** | **reject** |
| --- | --- | --- | --- | --- | --- | --- | --- |
| 1 | 0 ng L⁻¹ | 20 ng L⁻¹ | 12500 | 0.219 | -4267.69 | 29267.69 | FALSE |
| 1 | 0 ng L⁻¹ | 60 ng L⁻¹ | 15825 | 0.071 | -942.69 | 32592.69 | FALSE |
| 1 | 0 ng L⁻¹ | 90 ng L⁻¹ | 22500 | **0.005** | 5732.31 | 39267.69 | **TRUE** |
| 1 | 0 ng L⁻¹ | 120 ng L⁻¹ | 28150 | **0.001** | 11382.31 | 44917.69 | **TRUE** |
| 1 | 0 ng L⁻¹ | 200 ng L⁻¹ | 24150 | **0.003** | 7382.31 | 40917.69 | **TRUE** |
| 2 | 0 ng L⁻¹ | 20 ng L⁻¹ | 33300 | 0.118 | -5519.81 | 72119.81 | FALSE |
| 2 | 0 ng L⁻¹ | 60 ng L⁻¹ | 61650 | **0.001** | 22830.19 | 100469.8 | **TRUE** |
| 2 | 0 ng L⁻¹ | 90 ng L⁻¹ | 78325 | **0.000** | 39505.19 | 117144.8 | **TRUE** |
| 2 | 0 ng L⁻¹ | 120 ng L⁻¹ | 83325 | **0.000** | 44505.19 | 122144.8 | **TRUE** |
| 2 | 0 ng L⁻¹ | 200 ng L⁻¹ | 129150 | **0.000** | 90330.19 | 167969.8 | **TRUE** |
| 3 | 0 ng L⁻¹ | 20 ng L⁻¹ | 44175 | **0.000** | 18880.03 | 69469.97 | **TRUE** |
| 3 | 0 ng L⁻¹ | 60 ng L⁻¹ | 65050 | **0.000** | 39755.03 | 90344.97 | **TRUE** |
| 3 | 0 ng L⁻¹ | 90 ng L⁻¹ | 114225 | **0.000** | 88930.03 | 139520 | **TRUE** |
| 3 | 0 ng L⁻¹ | 120 ng L⁻¹ | 139200 | **0.000** | 113905 | 164495 | **TRUE** |
| 3 | 0 ng L⁻¹ | 200 ng L⁻¹ | 163375 | **0.000** | 138080 | 188670 | **TRUE** |
| 4 | 0 ng L⁻¹ | 20 ng L⁻¹ | 43325 | 0.185 | -12403.9 | 99053.88 | FALSE |
| 4 | 0 ng L⁻¹ | 60 ng L⁻¹ | 121650 | **0.000** | 65921.12 | 177378.9 | **TRUE** |
| 4 | 0 ng L⁻¹ | 90 ng L⁻¹ | 149175 | **0.000** | 93446.12 | 204903.9 | **TRUE** |
| 4 | 0 ng L⁻¹ | 120 ng L⁻¹ | 150000 | **0.000** | 94271.12 | 205728.9 | **TRUE** |
| 4 | 0 ng L⁻¹ | 200 ng L⁻¹ | 174150 | **0.000** | 118421.1 | 229878.9 | **TRUE** |
| 5 | 0 ng L⁻¹ | 20 ng L⁻¹ | 89975 | **0.000** | 42352.69 | 137597.3 | **TRUE** |
| 5 | 0 ng L⁻¹ | 60 ng L⁻¹ | 150800 | **0.000** | 103177.7 | 198422.3 | **TRUE** |
| 5 | 0 ng L⁻¹ | 90 ng L⁻¹ | 181625 | **0.000** | 134002.7 | 229247.3 | **TRUE** |
| 5 | 0 ng L⁻¹ | 120 ng L⁻¹ | 202475 | **0.000** | 154852.7 | 250097.3 | **TRUE** |
| 5 | 0 ng L⁻¹ | 200 ng L⁻¹ | 251675 | **0.000** | 204052.7 | 299297.3 | **TRUE** |
| 6 | 0 ng L⁻¹ | 20 ng L⁻¹ | 155000 | **0.000** | 109979.2 | 200020.8 | **TRUE** |
| 6 | 0 ng L⁻¹ | 60 ng L⁻¹ | 169850 | **0.000** | 124829.2 | 214870.8 | **TRUE** |
| 6 | 0 ng L⁻¹ | 90 ng L⁻¹ | 215000 | **0.000** | 169979.2 | 260020.8 | **TRUE** |
| 6 | 0 ng L⁻¹ | 120 ng L⁻¹ | 243350 | **0.000** | 198329.2 | 288370.8 | **TRUE** |
| 6 | 0 ng L⁻¹ | 200 ng L⁻¹ | 369150 | **0.000** | 324129.2 | 414170.8 | **TRUE** |

**Table S2:** PERMANOVA summary for fatty acid profile (w%) in *E. gracilis* at different concentrations of B_12_ (0ng L^-1^,20 ng L^-1^, 60 ng L^-1^, 90 ng L^-1^, 120 ng L^-1^, and 200 ng L^-1^).

| **Source of variation** | **df** | **SS** | **MS** | **Pesudo-F** | **Unique permes** | **p** |
| --- | --- | --- | --- | --- | --- | --- |
| B_12_ doses | 5 | 17.29 | 3.46 | 3.21 | 999 | **0.014** |
| Residual | 12 | 12.94 | 1.08 |  |  |  |
| Total | 17 | 30.22 |  |  |  |  |

**Table S3:** Descriptive statistics (mean and standard deviation) for fatty acid w% in *E. gracilis* at varying levels of B_12_ concentrations (0ng L^-1^, 20 ng L^-1^, 60 ng L^-1^, 90 ng L^-1^, 120 ng L^-1^, and 200 ng L^-1^).

|  | **14:0** | | **16:0** | | **16:1n-7** | | **16:2n-7** | | **16:1n-6** | |
| --- | --- | --- | --- | --- | --- | --- | --- | --- | --- | --- |
| **Treatments** | **Mean** | **SD** | **Mean** | **SD** | **Mean** | **SD** | **Mean** | **SD** | **Mean** | **SD** |
| 0 ng L⁻¹ | 2.87 | 0.17 | 15.78 | 0.92 | 3.80 | 0.51 | 2.33 | 0.24 | 3.19 | 0.02 |
| 20 ng L⁻¹ | 3.01 | 0.15 | 14.11 | 0.14 | 3.71 | 0.58 | 2.46 | 0.05 | 3.35 | 0.01 |
| 60 ng L⁻¹ | 2.96 | 0.13 | 14.28 | 0.41 | 3.71 | 0.59 | 2.40 | 0.14 | 3.33 | 0.16 |
| 90 ng L⁻¹ | 3.01 | 0.22 | 14.12 | 0.19 | 3.59 | 0.30 | 2.35 | 0.05 | 3.33 | 0.07 |
| 120 ng L⁻¹ | 2.78 | 0.01 | 13.62 | 0.62 | 4.03 | 0.19 | 2.57 | 0.08 | 3.23 | 0.08 |
| 200 ng L⁻¹ | 2.90 | 0.05 | 14.59 | 1.33 | 3.81 | 0.31 | 2.38 | 0.26 | 3.25 | 0.03 |
|  | **16:3n-6** | | **16:4n-3** | | **18:0** | | **18:1n-9** | | **18:1n-7** | |
|  | **Mean** | **SD** | **Mean** | **SD** | **Mean** | **SD** | **Mean** | **SD** | **Mean** | **SD** |
| 0 ng L⁻¹ | 0.54 | 0.02 | 11.31 | 0.20 | 0.53 | 0.06 | 2.54 | 0.15 | 2.22 | 0.10 |
| 20 ng L⁻¹ | 0.64 | 0.05 | 10.37 | 0.26 | 0.42 | 0.02 | 2.78 | 0.04 | 2.26 | 0.06 |
| 60 ng L⁻¹ | 0.58 | 0.01 | 9.85 | 0.31 | 0.47 | 0.03 | 2.75 | 0.04 | 2.34 | 0.12 |
| 90 ng L⁻¹ | 0.62 | 0.04 | 9.98 | 0.25 | 0.46 | 0.02 | 2.73 | 0.04 | 2.32 | 0.04 |
| 120 ng L⁻¹ | 0.64 | 0.02 | 10.50 | 0.12 | 0.43 | 0.02 | 2.76 | 0.09 | 2.38 | 0.03 |
| 200 ng L⁻¹ | 0.59 | 0.05 | 10.83 | 0.55 | 0.47 | 0.08 | 2.54 | 0.30 | 2.28 | 0.15 |
|  | **18:2n-6** | | **18:3n-3** | | **20:0** | | **20:2n-6** | | **20:4n-6** | |
|  | **Mean** | **SD** | **Mean** | **SD** | **Mean** | **SD** | **Mean** | **SD** | **Mean** | **SD** |
| 0 ng L⁻¹ | 3.59 | 0.28 | 23.34 | 0.24 | 1.37 | 1.00 | 2.18 | 0.13 | 4.07 | 0.32 |
| 20 ng L⁻¹ | 3.81 | 0.19 | 21.68 | 0.59 | 1.46 | 0.65 | 2.50 | 0.05 | 4.41 | 0.17 |
| 60 ng L⁻¹ | 3.87 | 0.24 | 21.88 | 0.38 | 2.21 | 0.53 | 2.46 | 0.01 | 4.51 | 0.04 |
| 90 ng L⁻¹ | 3.91 | 0.14 | 21.90 | 0.43 | 1.06 | 0.70 | 2.49 | 0.03 | 4.58 | 0.16 |
| 120 ng L⁻¹ | 3.96 | 0.09 | 21.64 | 0.54 | 1.39 | 0.18 | 2.51 | 0.11 | 4.58 | 0.27 |
| 200 ng L⁻¹ | 3.90 | 0.15 | 22.26 | 0.77 | 1.78 | 0.80 | 2.28 | 0.29 | 4.31 | 0.48 |
|  | **20:3n-3** | | **20:3n-6** | | **20:5n-3** | | **22:5n-3** | | **22:6n-3** | |
|  | **Mean** | **SD** | **Mean** | **SD** | **Mean** | **SD** | **Mean** | **SD** | **Mean** | **SD** |
| 0 ng L⁻¹ | 1.47 | 0.09 | 2.07 | 0.12 | 8.34 | 0.28 | 3.23 | 0.18 | 6.59 | 0.12 |
| 20 ng L⁻¹ | 1.74 | 0.10 | 2.50 | 0.22 | 8.72 | 0.36 | 3.85 | 0.06 | 7.68 | 0.18 |
| 60 ng L⁻¹ | 1.74 | 0.02 | 2.47 | 0.09 | 8.60 | 0.13 | 3.97 | 0.17 | 7.84 | 0.11 |
| 90 ng L⁻¹ | 1.73 | 0.08 | 2.47 | 0.13 | 8.75 | 0.15 | 3.90 | 0.02 | 7.78 | 0.22 |
| 120 ng L⁻¹ | 1.75 | 0.06 | 2.38 | 0.11 | 8.49 | 0.12 | 3.96 | 0.16 | 7.79 | 0.12 |
| 200 ng L⁻¹ | 1.60 | 0.15 | 2.30 | 0.14 | 8.48 | 0.13 | 3.76 | 0.41 | 7.48 | 0.38 |

**Table S4:** Descriptive statistics (mean and standard deviation) for fatty acid w% in *E. gracilis* at different B_12_ concentrations and nitrogen levels.

| **Treatments** | **Nitrogen** | **B_12_** | **14:0** | | **16:0** | | **16:1n-7** | | **16:2n-7** | | **16:1n-6** | |
| --- | --- | --- | --- | --- | --- | --- | --- | --- | --- | --- | --- | --- |
|  |  |  | **Mean** | **SD** | **Mean** | **SD** | **Mean** | **SD** | **Mean** | **SD** | **Mean** | **SD** |
| N+No B_12_ | Sufficient | 0 ng L⁻¹ | 3.25 | 1.04 | 22.05 | 5.42 | 2.74 | 1.37 | 3.84 | 0.72 | 2.80 | 0.64 |
| N deplete+NoB_12_ | Low | 0 ng L⁻¹ | 3.72 | 0.25 | 19.50 | 2.38 | 2.74 | 0.88 | 3.76 | 0.28 | 3.33 | 0.37 |
| N+B_12_ deplete | Sufficient | 9 ng L⁻¹ | 3.68 | 0.52 | 17.75 | 7.69 | 2.07 | 0.76 | 4.02 | 0.64 | 3.19 | 0.34 |
| (N+B_12_)deplete | Low | 9 ng L⁻¹ | 3.87 | 0.44 | 21.60 | 3.27 | 1.91 | 0.74 | 3.99 | 0.61 | 3.21 | 0.52 |
| Control/(N+B_12_) | Sufficient | 90 ng L⁻¹ | 3.76 | 0.36 | 20.05 | 1.55 | 1.81 | 0.78 | 4.03 | 0.67 | 3.44 | 0.81 |
| N deplete+ B_12_ | Low | 90 ng L⁻¹ | 3.49 | 0.14 | 19.30 | 0.87 | 2.36 | 0.96 | 3.65 | 0.20 | 3.38 | 0.27 |
|  |  |  | **16:3n-6** | | **16:4n-3** | | **18:0** | | **18:1n-9** | | **18:1n-7** | |
|  |  |  | **Mean** | **SD** | **Mean** | **SD** | **Mean** | **SD** | **Mean** | **SD** | **Mean** | **SD** |
| N+No B_12_ | Sufficient | 0 ng L⁻¹ | 1.28 | 0.19 | 1.04 | 0.21 | 1.99 | 1.18 | 2.77 | 0.15 | 3.75 | 0.42 |
| N deplete+NoB_12_ | Low | 0 ng L⁻¹ | 1.40 | 0.13 | 1.08 | 0.05 | 1.27 | 0.73 | 2.92 | 0.18 | 4.06 | 0.40 |
| N+B12 deplete | Sufficient | 9 ng L⁻¹ | 1.39 | 0.15 | 1.04 | 0.18 | 1.76 | 0.92 | 2.83 | 0.24 | 4.22 | 0.58 |
| (N+B12)deplete | Low | 9 ng L⁻¹ | 1.34 | 0.15 | 0.99 | 0.10 | 1.27 | 0.40 | 2.90 | 0.18 | 4.09 | 0.43 |
| Control/(N+B_12_) | Sufficient | 90 ng L⁻¹ | 1.36 | 0.32 | 1.17 | 0.14 | 1.21 | 0.33 | 2.91 | 0.29 | 4.08 | 0.58 |
| N deplete+ B_12_ | Low | 90 ng L⁻¹ | 1.47 | 0.10 | 1.06 | 0.10 | 1.25 | 0.37 | 2.89 | 0.23 | 4.23 | 0.18 |
|  |  |  | **18:2n-6** | | **18:3n-3** | | **20:0** | | **20:2n-6** | | **20:4n-6** | |
|  |  |  | **Mean** | **SD** | **Mean** | **SD** | **Mean** | **SD** | **Mean** | **SD** | **Mean** | **SD** |
| N+No B_12_ | Sufficient | 0 ng L⁻¹ | 4.38 | 0.41 | 28.51 | 1.14 | 2.11 | 1.06 | 3.24 | 0.40 | 4.09 | 0.47 |
| N deplete+NoB_12_ | Low | 0 ng L⁻¹ | 5.19 | 0.24 | 28.38 | 0.14 | 1.05 | 0.52 | 3.21 | 0.21 | 4.53 | 0.61 |
| N+B_12_ deplete | Sufficient | 9 ng L⁻¹ | 4.82 | 0.58 | 30.22 | 3.52 | 1.88 | 0.48 | 3.17 | 0.20 | 4.36 | 0.44 |
| (N+B_12_)deplete | Low | 9 ng L⁻¹ | 4.84 | 0.40 | 28.09 | 1.00 | 1.57 | 1.08 | 3.17 | 0.25 | 4.30 | 0.68 |
| Control/(N+B_12_) | Sufficient | 90 ng L⁻¹ | 4.17 | 1.94 | 29.24 | 1.90 | 1.36 | 0.64 | 3.37 | 0.24 | 3.91 | 1.79 |
| N deplete+ B_12_ | Low | 90 ng L⁻¹ | 4.77 | 0.24 | 28.52 | 1.00 | 1.29 | 0.15 | 3.15 | 0.25 | 4.42 | 0.30 |
|  |  |  | **20:3n-3** | | **20:3n-6** | | **20:5n-3** | | **22:5n-3** | | **22:6n-3** | |
|  |  |  | **Mean** | **SD** | **Mean** | **SD** | **Mean** | **SD** | **Mean** | **SD** | **Mean** | **SD** |
| N+No B_12_ | Sufficient | 0 ng L⁻¹ | 4.88 | 3.08 | 2.32 | 0.55 | 10.82 | 4.44 | 0.35 | 0.06 | 0.79 | 0.28 |
| N deplete+NoB_12_ | Low | 0 ng L⁻¹ | 4.84 | 2.34 | 2.33 | 0.29 | 11.40 | 1.01 | 0.32 | 0.06 | 0.84 | 0.12 |
| N+B_12_ deplete | Sufficient | 9 ng L⁻¹ | 5.67 | 3.26 | 2.37 | 0.39 | 11.92 | 1.36 | 0.35 | 0.02 | 0.83 | 0.09 |
| (N+B_12_)deplete | Low | 9 ng L⁻¹ | 6.12 | 3.15 | 2.31 | 0.49 | 11.04 | 1.76 | 0.30 | 0.09 | 0.79 | 0.15 |
| Control/(N+B_12_) | Sufficient | 90 ng L⁻¹ | 1.92 | 1.25 | 2.45 | 0.64 | 11.84 | 1.84 | 0.35 | 0.08 | 0.85 | 0.13 |
| N deplete+ B_12_ | Low | 90 ng L⁻¹ | 4.12 | 2.49 | 2.64 | 0.31 | 12.26 | 1.09 | 0.32 | 0.05 | 0.85 | 0.11 |

**Table S5:** Significance test ANOVA/Tukey familywise between control that is grown with exogenous B_12_ without adding bacteria and different bacteria-to-algae ratio (1:1, 5:1, 10:1, and 20:1). Significant factors are given in bold color.

| **Day** | ***p value*** | | | | |
| --- | --- | --- | --- | --- | --- |
|  | **90ng L⁻¹ VS 1:1 ratio** | **90ng L⁻¹ VS 5:1 ratio** | **90ng L⁻¹ VS 10:1 ratio** | **90ng L⁻¹ VS 20:1 ratio** |  |
| 1 | 0.919 | 0.209 | **0.006** | **0.000** | |
| 2 | 0.998 | **0.001** | **0.000** | **0.000** | |
| 3 | 0.947 | 0.984 | **0.050** | **0.000** | |
| 4 | 1.000 | 0.732 | 0.095 | **0.000** | |
| 5 | 0.996 | 0.851 | 0.218 | **0.000** | |
| 6 | 0.999 | 0.998 | 0.840 | **0.001** | |
| 7 | 1.000 | 0.990 | 0.987 | **0.027** | |
| 8 | 0.996 | 0.979 | 0.995 | **0.051** | |
| 9 | 0.995 | 0.920 | 0.994 | 0.078 | |
| 10 | 1.000 | 1.000 | 1.000 | 0.136 | |

**Table S6:** PERMANOVA summary for fatty acid profile (w%) in *E. gracilis* at control conditions that are grown with exogenous B_12_ without adding bacteria and different bacteria-to-algae ratios (1:1, 5:1, 10:1, and 20:1).

| Source of variation | df | SS | MS | Pesudo-F | Unique permes | p |
| --- | --- | --- | --- | --- | --- | --- |
| Bacteria-to-Algae ratio | 4 | 0.056 | 0.014 | 1.816 | 999 | **0.012** |
| Residual | 23 | 0.178 | 0.008 |  |  |  |
| Total | 27 | 0.234 |  |  |  |  |

**Table S7:** Descriptive statistics (mean and standard deviation) for fatty acid w% in *E. gracilis* at control that is grown with exogenous B_12_ without adding bacteria and different bacteria-to-algae ratio (1:1, 5:1, 10:1, and 20:1).

|  | **14:0** | | **16:0** | | **16:1n-7** | | **16:2n-7** | | **16:1n-6** | |
| --- | --- | --- | --- | --- | --- | --- | --- | --- | --- | --- |
| **Treatments** | **Mean** | **SD** | **Mean** | **SD** | **Mean** | **SD** | **Mean** | **SD** | **Mean** | **SD** |
| 90ng L⁻¹ B_12_ | 3.66 | 0.13 | 20.21 | 2.59 | 3.54 | 0.73 | 2.37 | 0.18 | 4.28 | 0.25 |
| 1:1 ratio | 3.20 | 0.12 | 16.90 | 0.75 | 3.64 | 0.42 | 2.31 | 0.11 | 4.47 | 0.20 |
| 5:1 ratio | 3.56 | 0.64 | 19.37 | 6.11 | 3.15 | 1.45 | 2.70 | 0.36 | 4.12 | 0.52 |
| 10:1 ratio | 3.29 | 0.27 | 16.73 | 0.56 | 3.69 | 0.37 | 2.52 | 0.21 | 4.45 | 0.22 |
| 20:1 ratio | 3.47 | 0.48 | 15.22 | 1.24 | 3.82 | 0.79 | 2.50 | 0.32 | 4.40 | 0.50 |
|  | **16:3n-6** | | **16:4n-3** | | **18:0** | | **18:1n-9** | | **18:1n-7** | |
|  | **Mean** | **SD** | **Mean** | **SD** | **Mean** | **SD** | **Mean** | **SD** | **Mean** | **SD** |
| 90ng L⁻¹ B12 | 0.85 | 0.14 | 11.17 | 0.53 | 1.23 | 0.66 | 2.19 | 0.11 | 2.49 | 0.13 |
| 1:1 ratio | 1.14 | 0.06 | 11.37 | 0.22 | 0.45 | 0.10 | 2.56 | 0.20 | 2.78 | 0.13 |
| 5:1 ratio | 1.02 | 0.15 | 11.10 | 1.60 | 0.63 | 0.33 | 2.33 | 0.07 | 2.63 | 0.23 |
| 10:1 ratio | 1.08 | 0.10 | 11.84 | 0.52 | 0.41 | 0.13 | 2.71 | 1.08 | 3.02 | 1.03 |
| 20:1 ratio | 1.14 | 0.14 | 11.33 | 1.19 | 0.40 | 0.10 | 2.22 | 0.23 | 2.54 | 0.31 |
|  | **18:2n-6** | | **18:3n-3** | | **20:0** | | **20:2n-6** | | **20:4n-6** | |
|  | **Mean** | **SD** | **Mean** | **SD** | **Mean** | **SD** | **Mean** | **SD** | **Mean** | **SD** |
| 90ng L⁻¹ B12 | 4.40 | 0.39 | 21.99 | 0.39 | 0.22 | 0.14 | 2.31 | 0.22 | 3.85 | 0.27 |
| 1:1 ratio | 4.95 | 0.41 | 20.58 | 0.53 | 0.08 | 0.03 | 3.10 | 0.21 | 5.10 | 0.43 |
| 5:1 ratio | 4.75 | 0.53 | 21.02 | 1.79 | 0.13 | 0.10 | 2.77 | 0.26 | 4.56 | 0.53 |
| 10:1 ratio | 4.72 | 0.44 | 21.68 | 0.62 | 0.09 | 0.05 | 2.58 | 0.17 | 4.27 | 0.22 |
| 20:1 ratio | 4.76 | 0.55 | 20.33 | 2.08 | 0.04 | 0.02 | 2.66 | 0.28 | 8.71 | 9.24 |
|  | **20:3n-3** | | **20:3n-6** | | **20:5n-3** | | **22:5n-3** | | **22:6n-3** | |
|  | **Mean** | **SD** | **Mean** | **SD** | **Mean** | **SD** | **Mean** | **SD** | **Mean** | **SD** |
| 90ng L⁻¹ B12 | 1.96 | 0.16 | 2.68 | 0.22 | 10.18 | 0.53 | 0.35 | 0.02 | 0.68 | 0.05 |
| 1:1 ratio | 2.12 | 0.06 | 3.19 | 0.08 | 10.87 | 0.20 | 0.41 | 0.02 | 0.78 | 0.01 |
| 5:1 ratio | 1.93 | 0.13 | 2.81 | 0.23 | 10.32 | 0.88 | 0.38 | 0.02 | 0.74 | 0.05 |
| 10:1 ratio | 2.13 | 0.21 | 2.88 | 0.20 | 10.77 | 0.48 | 0.38 | 0.02 | 0.76 | 0.04 |
| 20:1 ratio | 2.14 | 0.24 | 2.91 | 0.32 | 10.29 | 0.96 | 0.38 | 0.04 | 0.74 | 0.08 |

**Table S8:** OLS regression results, homogeneity of slopes test via ANOVA F-test, and post hoc pairwise slope difference t-test with Bonferroni (n=72) for algal cell density of *E. gracilis* when grown with and without DOC in the presence and absence of membrane.

**OLS Regression Results**

Dep. Variable: Q (OD 680)	R-squared: 0.9410

Model: OLS	Adj. R-squared: 0.9340

Method: Least Squares	F-statistic: 145.20

No. Observations: 72	AIC: -555.40

Df Residuals: 64	BIC: -537.20

Df Model: 7

Covariance Type: Nonrobust

| Pair | t-value | p-value |
| --- | --- | --- |
| TI vs. TII | 6.017 | <0.001 |
| TI vs. TIII | 0.821 | 0.415 |
| TI vs. TIV | 6.433 | <0.001 |
| TII vs. TIII | 5.196 | <0.001 |
| TII vs. TIV | 0.416 | 0.679 |
| TIII vs. TIV | 5.612 | <0.001 |

Note: Treatment I: *E. gracilis* + *M. loti* in membrane with DOC

Treatment II: *E. gracilis* + *M. loti* in membrane without DOC

Treatment III: *E. gracilis* + *M. loti* without membrane with DOC

Treatment IV: *E. gracilis* + *M. loti* without membrane without DOC
