# Supplementary material for: Vitamin B12 Availability and Interaction with Bacterium Mesorhizobium loti Affect Growth and Biochemical Composition of Mixotrophic Alga Euglena Gracilis
Source: Microb Ecol. 2026 Jun 23;89:169. doi: 10.1007/s00248-026-02816-0 (PMC13542019; doi:10.1007/s00248-026-02816-0)
Supplement: Supplementary file 2 [file 248_2026_2816_MOESM2_ESM.docx]

**Figure S1:** Scatter plot of algal cell density by spectrophotometer reading (OD 680) against hemocytometer cell counting (cells mL^-1^) at control conditions that are grown with exogenous B_12_ without adding different bacteria-to-algae ratios (1:1, 5:1, 10:1, and 20:1).

**Figure S2:** Cell density of *E. gracilis* increased with increasing B_12_ availability in the culture media. Error bars represent the standard errors (n=6).

**Figure S3:** The box plots of total fatty acid content (μg mg⁻¹ DW) in *E. gracilis* at different concentrations of B_12_ (0ng L^-1^, 20 ng L^-1^, 60 ng L^-1^, 90 ng L^-1^, 120 ng L^-1^, and 200 ng L^-1^). Boxes show IQR (interquartile range), with horizontal lines inside the box denoting the median, and lower and upper ends of whiskers represent minimum and maximum values. * denotes significance at p< 0.05.

**Figure S4:** The non-metric multidimensional (NMDS) plot of fatty acid profiles (w%) in treatments without B_12_ (0 ng L^-1^) and with varying levels of B_12_ (20 ng L^-1^, 60 ng L^-1^, 90 ng L^-1^, 120 ng L^-1^, and 200 ng L^-1^). Fatty acids, that correlate strongly (r >0.7) with either of the dimensions are presented. Vectors indicate the strength and direction of the correlation between fatty acids and the dimension.

**Figure S5:** The non-metric multidimensional (NMDS) plot of fatty acid profiles (w%) under varying levels of nitrogen and B_12_. Fatty acids that correlate strongly (r>0.6) with either dimension are presented. Vectors indicate the strength and direction of the correlation between fatty acids and the dimension.

**Figure S6:** The non-metric multidimensional (NMDS) plot of fatty acid profiles (w%) of the control that is grown with exogenous B_12_ without adding bacteria and different bacteria-to-algae ratios (1:1, 5:1, 10:1, and 20:1). Fatty acids that correlate strongly (r>0.7) with either dimension are presented. Vectors indicate the strength and direction of the correlation between fatty acids and dimension.

**Figure S7:** The box plots of total fatty acid content (μg mg⁻¹ DW) in *E. gracilis* at control conditions that are grown with exogenous B_12_ without adding different bacteria-to-algae ratios (1:1, 5:1, 10:1, and 20:1). Boxes show IQR (interquartile range), with horizontal lines inside the box denoting the median, and lower and upper ends of whiskers represent minimum and maximum values * denotes *p<0.05,* and *** denotes *p<0.001* when compared with 90 ng L⁻¹ B12.

**FigureS8:** Scatter plot of cellular B₁₂ content (OH-Cbl; nmol g^-1^) against the specific growth rate for control (90 ng L^-1^B_12_) and different bacteria-to-algae ratios (1:1, 5:1, 10:1, and 20:1). Number of replicates differ between treatments from n=3 to n=5.

**Figure S9:** Bar graph showing different variants of B_12_ produced by *M. loti*

**Figure S10:** Growth of *M. loti* measured as optical density (OD 580) over 72 hours.

**Figure S11:** Flow cytometry detection of phagocytic uptake of *M. loti* by *E. gracilis.* *M. Loti* were stained with 5 uM DAPI for 30 min, and after extensive washing, incubated for 4 hours with *E. gracilis*. As a negative control, unstained bacteria were used. After washing, fluorescence of *E. gracilis* was quantified by flow cytometry, (A) Gating of algae cells, (B) Relative fluorescent signal of algae cells after incubation with labelled and non-labelled bacteria (exc. 405 nm, 450 nm, peak fluorescence), and (C) Statistica mean for average and peak fluorescence in algal cells incubated with bacteria. A clear increase in fluorescence of algae was observed with labelled bacteria, suggesting phagocytosis of bacteria by the algae. This increase was not inhibited by 20 μM cytochalasin B, suggesting that either algal phagocytosis cannot be stopped with this inhibitor of actin polymerization or that the DAPI dye diffuses from bacterial cells into algae.
